# Circulating pro-angiogenic cells are preserved despite myocardial angiogenic signal deficits in HFpEF model

**DOI:** 10.1038/s41598-026-47557-9

**Published:** 2026-04-14

**Authors:** Pallavi Shrivastava, Rukayat A. Raji, Abdallah Jwayyed, Huijing Xia, Marian E. Okon, Ifechukwude J. Biose

**Affiliations:** 1 Cardiovascular Center of Excellence, Department of Pharmacology and Experimental Therapeutics, Louisiana State University Health Sciences Center, New Orleans, 70112 LA USA; 2 School of Graduate Studies, Louisiana State University Health Sciences Center, New Orleans, 70112 LA USA; 3 Department of Public Health Sciences, Xavier University of Louisiana, New Orleans, 70125 LA USA

**Keywords:** HFpEF, ZSF-1 obese rat, Endothelial progenitor cells, Angiogenic T-cells, Tang cells, Myocardial angiogenesis, Cardiology, Diseases, Medical research

## Abstract

Heart failure with preserved ejection fraction (HFpEF) has no cure, and microvascular dysfunction is a major contributing factor to its pathophysiology. Circulating endothelial progenitor cells (EPCs) and Angiogenic T (i.e., Tang) cells which support vascular repair are deficient in clinical HFpEF. The lack of data on circulating EPC and Tang cell counts in HFpEF models is an unmet critical gap in knowledge for potential therapeutic and biomarker development for HFpEF. In the clinically relevant HFpEF model (i.e., ZSF-1 obese rats), we hypothesized that circulating EPCs and Tang cell counts are significantly decreased compared to healthy control (WKY) rats. Thirty-eight-week-old male ZSF-1 obese and WKY rats were subjected to comprehensive phenotyping protocol, including echocardiography, exercise tolerance, aortic pressure measurement, and left ventricular diastolic function assessment. Blood was analyzed by flow cytometry for early (CD146⁺/CD34⁺/CD133⁺) and late (CD34⁺/CD146⁺) EPCs, as well as Tang cells (CD3⁺/CXCR4⁺/CD31⁺). Left ventricular expression of angiogenic genes (SDF1α, VEGFα) and pro-inflammatory IL-1β gene was quantified. ZSF-1 obese rats exhibited severe HFpEF phenotype with impaired diastolic function, elevated filling pressures, and significant exercise intolerance. Contrary to our expectations, circulating EPC and Tang cell counts in ZSF-1 obese rats are comparable to those of WKY rats whereas myocardial expression of SDF-1α and VEGF-α were markedly reduced and IL-1β gene increased. These findings suggest a novel dissociation between circulating and tissue angiogenic profiles in HFpEF. Preserved circulating pro-angiogenic cells despite myocardial angiogenic deficits suggests angiogenic dissociation as a potential mechanism underlying microvascular dysfunction in HFpEF. While ZSF-1 lean rats would have been the ideal control instead of WKY, targeting myocardial angiogenic pathways in HFpEF may represent a promising therapeutic strategy.

**Supplementary Information:**

The online version contains supplementary material available at 10.1038/s41598-026-47557-9.

## Introduction

Heart failure with preserved ejection fraction (HFpEF) is a major clinical challenge, accounting for nearly half of all heart failure cases^1–3^ which disproportionately affect older adults with obesity, diabetes, and hypertension^4–6^. HFpEF arises mainly from diastolic stiffness, microvascular dysfunction, and systemic inflammation^7–10^. Despite its rising prevalence, there is no established cure, and therapies targeted at improving outcomes remain limited. This underscores the urgent need to pursue novel mechanistic perspectives for mitigating HFpEF.

Endothelial dysfunction and microvascular inflammation are at the center of HFpEF pathogenesis and they are the primary drivers of myocardial remodeling^11,12^. In this context, comorbidities such as obesity and hypertension promote a pro-inflammatory milieu which disrupts endothelial nitric oxide signaling, reduces capillary density, and triggers interstitial cardiac fibrosis. The resulting loss of microvascular integrity impairs oxygen delivery and diastolic ventricular relaxation, culminating in increased left ventricular filling pressures despite preserved systolic function. However, the cellular and molecular mechanisms linking cardiometabolic stress to impaired angiogenesis and microvascular rarefaction in HFpEF remain incompletely understood.

Circulating endothelial progenitor cells (EPCs) and angiogenic T-cells (also known as Tang cells) are key mediators of vascular repair and neovascularization^13,14^. Circulating EPCs contribute to endothelial regeneration through paracrine release of proangiogenic factors and direct endothelial incorporation, while Tang cells facilitate EPC mobilization and vascular homeostasis^15^. Clinical studies reported significantly low EPC and Tang cell counts in patients with HFpEF^16–18^ yet another distinct study found no difference in EPC counts between HFpEF and healthy control patients^19^. Even with this inconsistent observation, there exist no reports on EPC and Tang cells counts in experimental HFpEF models, and their relationship with myocardial angiogenic gene expression has not been defined in preclinical models.

The ZSF-1 obese rat, derived from a cross between the Zucker diabetic fatty (ZDF) and spontaneously hypertensive heart failure (SHHF) strains^20^, recapitulates many of the metabolic, hemodynamic, and structural abnormalities observed in human HFpEF, including obesity, hypertension, diastolic dysfunction, and exercise intolerance^22–25^. Therefore, ZSF-1 obese rat is a clinically relevant model of HFpEF with disease onset observed from 10 weeks of age^21,22,26^. However, the interplay between systemic angiogenic cell populations and local myocardial angiogenic signaling in this model has not been thoroughly characterized.

In this study, we characterized the phenotypic, hemodynamic, and angiogenic features of 38-week-old ZSF-1 obese rats compared with age-matched Wistar Kyoto (WKY) controls. We hypothesized that 38 weeks old male ZSF-1 obese rats with HFpEF would exhibit significantly reduced counts for circulating EPCs and Tang cells which will be accompanied by impaired myocardial expression of angiogenic factors when compared to age-matched healthy control Wistar Kyoto (WKY) rats. Given that HFpEF severity is noted from 20 weeks of age, we chose 38 weeks of age to capture the advanced phase of HFpEF severity which is similar to peri-hospital stage of HFpEF cases with impaired physical activity. While WKY rats do not share genetic background with ZSF-1 obese rats and they are considered healthy, non-hypertensive and lean control.

## Materials and methods

### Animal source and housing

At 38 weeks old, a total of 6 male WKY and 8 male ZSF-1 Obese rats (Charles River Laboratories, Wilmington, MA) were used for this study. Rats of similar genetic strain were housed in groups of two in a room with ambient temperature and humidity control. Animals had unlimited access to food and water and were conditioned to 12-h light/dark cycle. The experiments reported herein were approved by the Institutional Animal Care & Use Committee (IACUC) of the Louisiana State University Health Sciences Center -New Orleans under protocol #7255. All in vivo measurements were performed in accordance with guidelines of the National Institutes of Health https://olaw.nih.gov/policies-laws/phs-policy.htm. The procedures and results are reported in accordance with the ARRIVE guidelines https://arriveguidelines.org/arrive-guidelines.

### Exercise tolerance test

To assess overall cardiovascular function and exercise tolerance, we used rodent treadmill (IITC, Woodland Hills, California) as previously described^27^. Briefly, all rats were allowed 5 min of treadmill familiarization and adaptation to the test room. Following acclimation, rats were subjected to a 5-min warmup run at 0º incline with a starting speed of 6 m/min which gradually increased by 1.5 m/min until a final speed of 12 m/min was attained and maintained for the last 1 min. Exercise tolerance test with a speed of 12 m/min was initiated immediately after the warmup run. The treadmill inclination was maintained at 0º, due to an anticipated running difficulty for ZSF-1 obese rats overtime. Exhaustion was indicated by a rat’s inability to run after three consecutive shocks within 15 s. Running duration was recorded and used to calculate exercise distance in meters.

#### Echocardiography

Transthoracic echocardiography with Vevo 3100 echocardiography system (FUJIFILM VisualSonics Inc., Canada) was performed under isoflurane anesthesia (5% for induction and 2–3% for maintenance) carried in 1 L/min of 100% oxygen, with slight modifications from previously established protocol^22^. Briefly, under deep anesthesia and following the removal of ventral chest fur using Nair, pre-warmed ultrasound gel was applied to the ventral thoracic region for image acquisition. Multiple parasternal long-axis M-mode images were taken from the base to the apex of the heart, as well as short-axis images of the left ventricle. Measurements of cardiac structure included interventricular septal wall thickness (IVSd, IVSs), left ventricular posterior wall thickness (LVPWd, LVPWs), and left ventricular diameters (LVEDD, LVESD). Percentage LV fractional shortening (FS) and LV ejection fraction (EF) were automatically calculated using Vevo LAB ultrasound analysis software 5.11.0 (https://www.visualsonics.com/product/software/vevo-lab).

Pulse wave Doppler echocardiography at the level of the mitral valve orifice was acquired to calculate transmitral inflow velocities during diastole (relaxation). Non-invasive diastolic function was calculated using the ratio of peak early (E) and late (A) transmitral velocities. Additionally, a clinically relevant index of diastolic function was calculated using the ratio of (E) to early tissue velocity (e’), where e’ at the level of the mitral valve is measured by pulse wave tissue Doppler. All images were analyzed using Vevo LAB ultrasound analysis software 5.11.0.

#### Invasive hemodynamics and blood collection

Invasive hemodynamics was performed under 2% isoflurane anesthesia using 2.0 -F transonic solid-state pressure transducer catheter (Millar, USA), immediately prior to euthanasia, with slight modifications from previously described methods^28,29^. The pressure catheter was surgically advanced to the root of the aorta through the right common carotid artery. Records of aortic systemic blood pressure were taken for a minimum of 30 s. Left ventricular systolic and diastolic blood pressure, LVEDP and the relaxation constant (Tau) were recorded following the advancement of the catheter into the left ventricle through the aortic valve. Data was acquired and analyzed using LabChart analysis software 8 (AD Instruments, USA; https://www.adinstruments.com/products/labchart?creative=783289317240&keyword=labchart&matchtype=p&network=g&device=c&gad_source=1&gad_campaignid=23246163986&gbraid=0AAAAADiCoTa1cBJRNrMt9RZBVOvtnSEK&gclid=Cj0KCQjw4a3OBhCHARIsAChaqJMzHsZmK-hYWQ_WGoemGvsmf4Pgwtbc6uoP94_8DD63FEr0qZKl9kaAmelEALw_wcB).

After withdrawing the pressure transducer catheter from the aorta, a bent 20G needle attached to 10 mL syringe was advanced into the aorta to collect ~ 10 mL of whole blood into EDTA coated container (BD Vacutainer; SKU:366,643) for the flow cytometry protocol described below.

### Isolation of peripheral blood mononuclear cells (PBMCs)

PBMCs were isolated using density gradient centrifugation with Ficoll-Paque PLUS (GE17-1440–02, Sigma) as previously described^19^. Briefly, whole blood was diluted 1:1 with phosphate-buffered saline (PBS) without calcium and magnesium (Gibco, Thermo Fisher Scientific). In 15 ml tubes and at room temperature, 8 ml of diluted blood was carefully added to 4 ml Ficoll density gradient medium and subjected to centrifugation at 1500 rpm for 30 min. The layer of buffy coat containing PBMCs was collected and washed twice with PBS by centrifugation at 3000 rpm for 8 min.

### EPCs and tang cell counts

Approximately 1 × 10^6^ cells/mL of PBMCs in separate tubes were allotted for quantifying EPCs and Tang cells. PBMCs were incubated with blocking reagent (i.e., 2% BSA with 0.05% Triton-X and 0.02% Tween-20) for 30 min. For each antibody per animal, single-stain vials of cells were created to contain each antibody (i.e., fluorophore) in addition to an experimental vial which is a cocktail of all antibodies (i.e., fluorophores) to simultaneously analyze multiple markers. This single-stain vial served as control to calculate compensation. The cells were incubated with fluorochrome-conjugated antibodies (1:100 in FACS buffer) for 60 min at room temp in the dark. Antibodies used wereanti-CD45 Spark Violet423 (BL202225), anti-CD31 BV605(BD744359), anti-CD34 PECY7(NBP2-33076PECY7), anti-CD133-AF488 (NB120-16518AF488), anti-CD146 (AF647), anti-CD31 BV605 (BD744359), anti-CD3 PECY5 (BL201419), and anti-CXCR4 AF700 (NBP1-77067AF700). Appropriate isotype controls (matched for antibody class, fluorochrome, and concentration) were included for each antibody to determine non-specific binding. Cells were washed twice with FACS buffer after incubation.

Gating for live circulating EPCs and Tang cells were done to distinguish lymphocytes from monocytes using the intensity of CD45 expression CD45^High^ expression marks lymphocytes while CD45^Medium^ generally marks monocytes. Fixable eFluro 780 was used for estimating live and dead cell populations. Single color profiles for different fluorochromes were used as controls. Multicolor flow cytometry was performed using Cytek Northern Lights system (NL-3000, Cytek Biosciences, Inc. Fremont, CA). This Cytek equipment has capabilities for three laser configurations: 405 nm: 100 mW, 488 nm: 50 mW, 640 nm: 80 mW. The violet detector module has 16 channels with uneven spaced bandwidth from 420–829 nm; blue detector module has 14 channels with uneven spaced bandwidth from 498–829 nm and the red detector module has 8 channels with uneven spaced bandwidth from 652–829 nm. Flow cytometer setup and calibration were performed using SpectroFlo QC Beads (cat- B7-10,001, Cytek) from wavelength 355 to 650 nm. Cytek SpectroFlo QC beads were used for routine performance tracking of Cytek flow cytometer NL-3000. A minimum of 100,000 events were acquired for P1 gate and flow rate is kept low at 15 µl/min. Spectral unmixing with autofluorescence extraction was done with stained and unstained cells.

Data analysis was performed using SpectroFlo Spectrum Cytometry software 3.3.0 (12,282,023; Cytek Biosciences, Inc. Fremont, CA; https://cytekbio.com/pages/aurora?utm_campaign=SO-Brand&utm_medium=cpc&utm_source=google&utm_term=cytek%20flow%20cytometry&utm_matchtype=b&gad_source=1&gad_campaignid=18900962336&gbraid=0AAAAACTxieSKQ_hpBX30FZhFIurEnhqsZ&gclid=Cj0KCQjw4a3OBhCHARIsAChaqJNM5hOCsUDs8ZqAmFR8Wm4V1v_tjewFu63QWt9Gd5Bri9DZnFOoDH4aAlBvEALw_wcB). Single cells were gated based on forward and side scatter characteristics. Compensation was performed using single-stained controls. EPC populations are defined as cells positive for endothelial markers (CD31^+^ and/or CD146^+^) as well as progenitor markers (CD34^+^ and/or CD133^+^). Consequently, early EPCs are defined as CD146^+^/CD34^+^/CD133^+^ while late EPCs are defined as CD34^+^/CD146^+^. Tang cell population are defined as CD31^+^/CD3^+^/CXCR4^+^. The percentage of cells expressing each marker and the co-expression of multiple markers were analyzed. Gates for positive and negative populations were determined based on isotype controls.

#### Quantitative polymerase chain reaction (Q-PCR)

Gene expression levels of stromal cell-derived factor 1α (SDF-1α), vascular endothelial growth factor α (VEGF-α) and pro-inflammatory cytokine interleukin-1β (IL-1β) were determined by reverse transcription polymerase chain reaction (RT-PCR; SYBR green assay). The housekeeping gene used was 18S rRNA. We focused on the SDF-1/CXCR4 axis and VEGF signaling since these pathways are central regulators of capillary rarefaction-induced angiogenesis^30–32^. SDF-1α was included to assess progenitor cell recruitment and regenerative angiogenesis while VEGF- α was selected as the primary driver of endothelial proliferation and vessel sprouting. Together, these markers provide a targeted evaluation of myocardial angiogenic transcription, unlike fibrotic markers (e.g., TGF-β, collagen) or pro-inflammatory cytokines (e.g., TNF-α, IL-6).

The sequences of the forward and reverse primers for each gene are shown in Table 1. Total RNA was extracted from the left ventricular tissue of ZSF-1 obese and WKY rats using RNeasy purification kit (Qiagen, Valencia, CA). The left ventricular tissue was homogenized in 1 mL of QIAzol Lysis Reagent (Cat-79306) using a Qiagen TissueLyser II with metal beads. Following homogenization, 200 µL of chloroform was added, and the mixture was centrifuged at 13,200 rpm for 20 min to separate the phases. The aqueous supernatant was collected, and an equal volume of 70% ethanol was added. Total RNA was then isolated from this mixture using the RNeasy purification kit with RW1 and RPE wash buffers. RNA purity and concentration were determined using a NanoDrop-1000 spectrophotometer (Thermo Scientific). Subsequently, 5 µg of the isolated RNA was converted into first-strand complementary DNA (cDNA) using the high-capacity RNA to cDNA EcoDry premix reverse transcription kit (cat- 639,548 Takara Bio USA, Inc). The cycling parameters were as follows: initial denaturation at 95 °C for 10 min followed by 45 cycles of 95 °C for 30 s, 60 °C for 60 s, and 72 °C for 30 s in Applied Biosystems StepOnePlus™ Real-Time PCR System (ABI, Carlsbad, CA). For each gene per animal, n = 3 technical replicates were performed and averaged. Relative quantity of mRNA was calculated by relating the PCR threshold cycle obtained from the tested sample to relative standard curves generated from a serial dilution of cDNA prepared from total cDNA and then quantified as a ratio of 18S rRNA.

**Table 1:** Rat primer sequences for RT-PCR.

Gene	Forward primer	Reverse primer
SDF-1α	CAGAGCCAACGTCAAGCA	AGGTACTCTTGGATCCAC
VEGF-α	GAAAAATTCACTGTGAGCCTTGTTC	CTTGGCTTGTCACATCTGCAA
IL-1β	CTCAATGGACAGAACATAAGCC	GGTGTGCCGTCTTTCATCA
18S rRNA	CATTCGAACGTCTGCCCTAT	GTTTCTCAGGCTCCCTCTCC

### Statistical analysis

Statistical analysis was performed using GraphPad Prism version 10.6.1 (892). Data was analyzed between groups with Welch’s t-test and summarized as mean ± standard deviation (SD). Also, simple linear regression analysis was performed to compare the relationships between variables. Outliers were identified using Grubb’s test. Statistical significance was set at p > 0.05.

## Results

### Phenotypic characteristics of severe HFpEF and healthy control rats

**Fig. 1:**
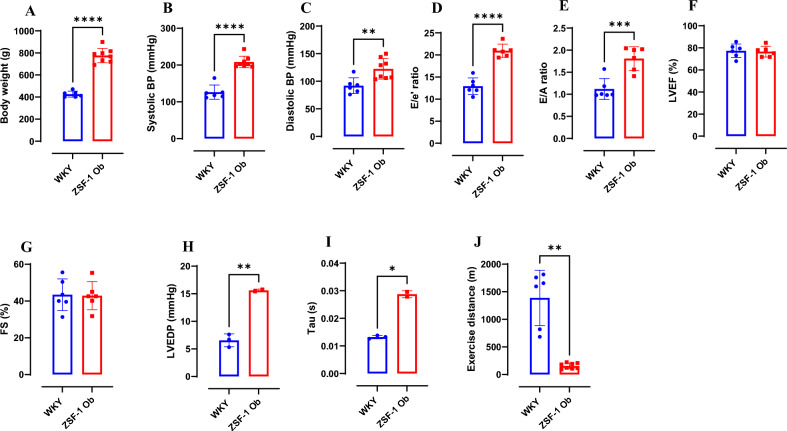
Phenotypic characteristics of severe HFpEF and healthy control rats. (**A**) Body weight (g), (**B**) Systolic blood pressure (mmHg), (**C**) Diastolic blood pressure (mmHg), (**D**) E/e’ ratio, (**E**) E/A ratio, (**F**) Left ventricular ejection fraction (%), (**G**) Fractional shortening (%), (**H**) Left ventricular end-diastolic pressure (mmHg), (**I**) Left ventricular relaxationconstant- Tau (s), (**J**) Exercise distance. Data expressed as mean ± SD. * P < 0.05, ** P < 0.01, *** P < 0.001; **** P < 0.0001. WKY: Wistar Kyoto rats; ZSF-1 Ob: ZSF-1 Obese rats.

**Fig. 2:**
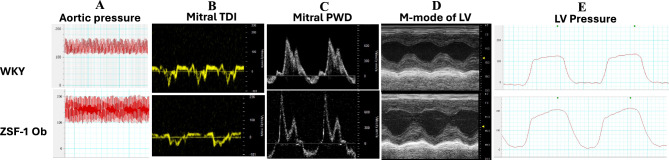
Representative images and plots from echocardiography and invasive hemodynamics of severe HFpEF and healthy control rats. (**A**) Aortic pressure plot acquired from invasive hemodynamics measurement, (**B**) Pulse wave Tissue Doppler imaging (TDI) acquired at the septal corner of the mitral annulus (**C**), Mitral inflow pulse wave Doppler (PWD) echocardiography, (**D**) M-mode of left ventricle (LV) acquired in short axis, (**E**) LV pressure plot acquired from invasive hemodynamics measurement. Top row represents images and plots from WKY (Wistar Kyoto rats), bottom row represents images and plots from ZSF-1 Obese (ZSF-1 Ob) rats.

At 38 weeks of age, ZSF-1 obese rats exhibit significantly higher body weight compared to age-matched WKY rats (p < 0.0001; Fig. 1A). Both systolic (p < 0.0001) and diastolic (p < 0.01) blood pressures were significantly elevated in ZSF-1 obese rats relative to WKY controls (Figs. 1B,C and 2A), confirming the obese and hypertensive phenotype characteristic of the HFpEF model.

Echocardiographic analysis revealed a significant increase in the E/e′ ratio (p < 0.0001; Figs. 1D and 2B) and E/A ratio (p < 0.001; Figs. 1E and 2C) in ZSF-1 obese relative to WKY rats, indicating elevated left ventricular (LV) filling pressures and restrictive filling from left ventricular stiffness, respectively. Despite these diastolic abnormalities, preserved LV ejection fraction (65–85%; p = 0.825; Figs. 1F and 2D) and fractional shortening (30–58%; p = 0.918; Figs. 1G and 2D) were observed to be statistically comparable between ZSF-1 obese and WKY rats. This indicates preserved systolic function.

Hemodynamic measurements further confirmed diastolic dysfunction in ZSF-1 obese rats, demonstrated by elevated LV end-diastolic pressure (p < 0.01; Figs. 1H and 2D) and prolonged myocardial relaxation constant (Tau; p < 0.05; Figs. 1I and 2D). In addition, ZSF-1 obese rats exhibited severe exercise intolerance as compared with WKY controls (p < 0.01; Fig. 1J). This was measured by total running distance on the treadmill and results were consistent with “exercise work” which captures difference in body weight between ZSF-1 obese and WKY rats, hence this result is not shown. “Exercise work” was assessed by running distance (m) X body weight (kg). Collectively, these findings confirm the presence of a severe HFpEF phenotype in 38-week-old ZSF-1 obese rats.

### Circulating angiogenic markers are comparable between ZSF-1 obese and WKY rats

**Fig. 3:**
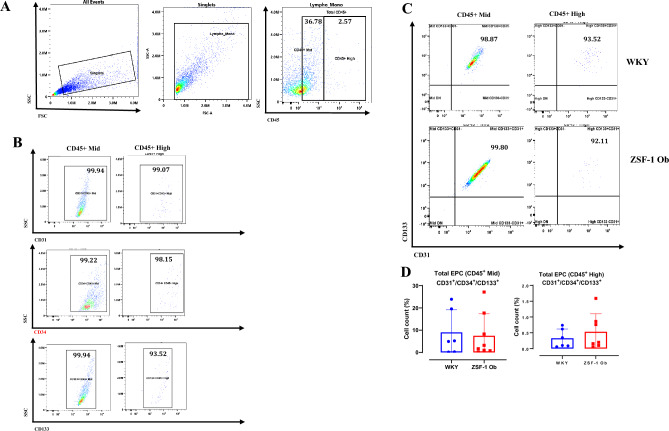
Comparable circulating overall endothelial progenitor cell (EPC) counts in ZSF-1 Ob and WKY rats. (**A**) Gating strategy for isolating peripheral blood mononuclear cells (PBMCs) using CD45 + antibodies for medium and high CD45 + expressing cells. (**B**) Single channels for identifying overall EPCs (CD31 +/CD34 +/CD133 +) and their representative plots as a subpopulation of CD45 + cells, (**C**) Representative plots for double staining of CD133 and CD31 for WKY rats (upper panel) and ZSF-1 Obese (lower panel), (**D**) Quantification of circulating overall EPCs as a percentage of medium and high CD45 + cells. Data expressed as mean ± SD.

**Fig. 4:**
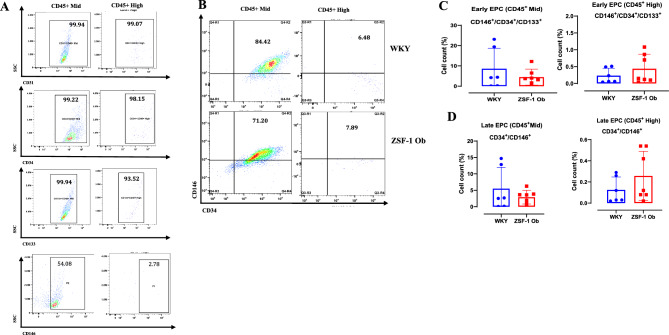
No difference in circulating early and late endothelial progenitor cell (EPC) counts between ZSF-1 Ob and WKY rats. (**A**) Single channels for identifying early EPCs (CD146 +/CD34 +/CD133 +) and late EPCs [CD146 +/CD34 +], (**B**) Representative plots of early and late EPC as a subpopulation of CD45 + cells for WKY rats (upper panel) and ZSF-1 Obese rats (lower panel), (**C**) Quantification of circulating early EPCs as a percentage of medium and high CD45 + cells, (**D**) Quantification of circulating late EPCs as a percentage of medium and high CD45 + cells. Data expressed as mean ± SD. One outlier excluded from ZSF-1 Ob group.

EPC populations did not differ significantly between ZSF-1 obese and WKY rats (Fig. 3). The proportion of general EPCs (CD31⁺/CD34⁺/CD133⁺) was comparable between groups for both CD45^Medium^ and CD45^High^ cell subsets (p = 0.7835 and p = 0.442, respectively; Fig. 3D). Similarly, circulating early EPCs (CD146⁺/CD34⁺/CD133⁺) and late EPCs (CD146⁺/CD34⁺) showed no significant differences between ZSF-1 obese and WKY rats for either CD45^Medium^ or CD45^High^ subsets (early EPCs: p = 0.338 and p = 0.319; late EPCs: p = 0.341 and p = 0.238; Fig. 4C,D).

**Fig. 5:**
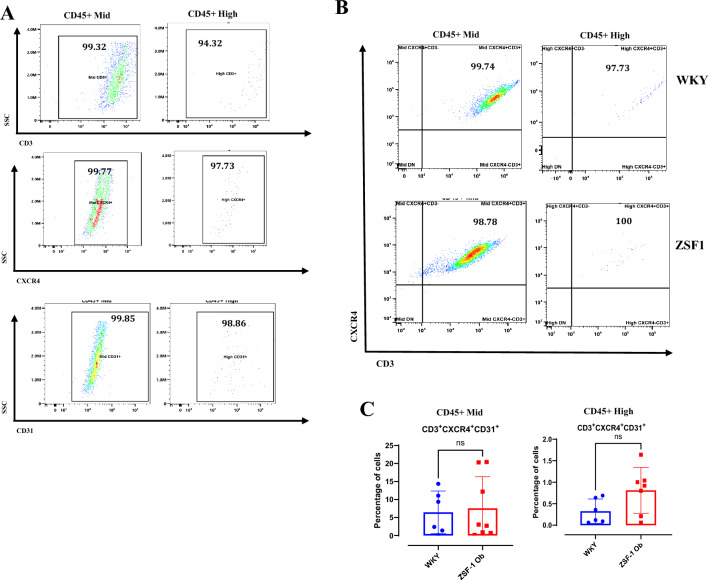
Tang cell count (i.e., Angiogenic T-cells) is not significantly different between ZSF-1 Ob and WKY rats. (**A**) Single channels for identifying Tang cells using CD3 +/CXCR4 +/CD31 + antibody markers within the population of medium and high CD45 + cells. (**B**) Representative plots of Tang cells for WKY rats (upper panel) and ZSF-1 Obese rats (lower panel), (**C**) Quantification of Angiogenic Tang cells as a percentage of medium and high CD45 + cells. Data expressed as mean ± SD. One outlier excluded from ZSF-1 Ob group.

Consistent with these findings, the percentage of circulating Tang cells (CD31⁺/CXCR4⁺/CD3⁺) was not significantly different between ZSF-1 obese and WKY rats (p = 0.800 and p = 0.073 for CD45^Medium^ and CD45^High^ subsets; Fig. 5C). These results indicate that circulating Tang cell populations are largely preserved in ZSF-1 obese rats despite marked HFpEF pathology.

For each circulating angiogenic cell type, our post hoc power calculations indicate that a minimum of n = 685 rats per group is required to detect statistically significant differences between groups. In the two-tailed analyses, α was set at 0.05 and power at 0.8. This suggest that the observed lack of differences may be biological but significantly underpowered.

### Decreased expression of angiogenic genes in the left ventricular tissue of ZSF-1 obese rats

**Fig. 6:**
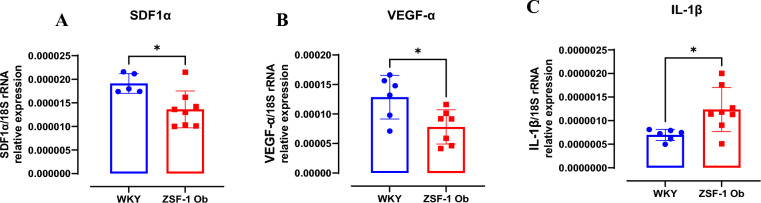
Myocardial angiogenic genes are low and a pro-inflammatory gene is significantly expressed in ZSF-1 obese compared to WKY rats. (**A**) Left ventricular gene expressions for (A) SDF-1α, (**B**) VEGF-α, (**C**) IL-1β. Data expressed as mean ± SD. * P < 0.05. WKY: Wistar Kyoto rats; ZSF-1 Ob: ZSF-1 Obese rats. One outlier excluded from WKY group.

**Fig. 7:**
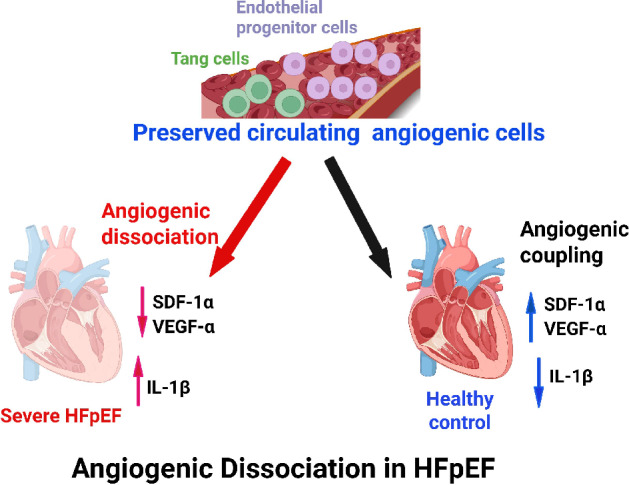
Summary schematic. Preserved circulating endothelial progenitor cells and Tang cells despite reduced myocardial SDF-1α, VEGF-α and increased IL-1β genes suggest angiogenic dissociation in severe HFpEF. This hints that impaired ventricular SDF-1α and VEGF-α signaling may contribute to microvascular dysfunction in HFpEF progression and warrants mechanistic investigations as potential therapeutic targets.

The Q-PCR analysis of left ventricular tissue revealed significantly reduced expression of SDF-1α (p < 0.05; Fig. 6A) and VEGF-α (p < 0.05; Fig. 6B) in ZSF-1 obese rats compared with WKY controls. Conversely, the expression of IL-1β was significantly upregulated in ZSF-1 obese rats (p < 0.05; Fig. 6C). Together, these findings suggest suppressed angiogenic signaling and increased pro-inflammatory activity in the myocardium of ZSF-1 obese rats with HFpEF (Fig. 7).

To assess the relationship between functional impairment and angiogenic dissociation, we performed a correlation analysis between exercise distance and myocardial SDF-1α gene expression as well as between exercise distance and E/e’ ratio. Exercise distance is positively correlated with SDF-1α but without statistical significance (*F*(1, 12) = 1.057, *p* = 0.324), R^2^ = 0.081; Supplementary Fig. 1 A). However, exercise distance is negatively correlated with E/e’ ratio with statistical significance (*F*(1, 10) = 46.34, *p* < 0.0001), R^2^ = 0.823, Supplementary Fig. 1B). These suggest that SDF-1α gene, but not ventricular filling pressure, increases as exercise capacity. In addition, to evaluate the relationship between circulating angiogenic cells and myocardial SDF-1α and VEGF-α gene expression, we conducted a correlation analysis between these cells and genes (Supplementary Fig. 2). Ventricular SDF-1α gene shows no correlation with either EPC count (*F*(1, 11) = 0.038, *p* = 0.85), R^2^ = 0.0034; Supplementary Fig. 2 A) nor with Tang cell counts (*F*(1, 12) = 0.0002, *p* = 0.99), R^2^ = 0.00002; Supplementary Fig. 2B). Also, ventricular VEGF-α gene shows statistically non-significant positive correlation with EPC counts (*F*(1, 11) = 0.9, *p* = 0.363), R^2^ = 0.076; Supplementary Fig. 2 C) and Tang cells (*F*(1, 11) = 0.65, *p* = 0.44), R^2^ = 0.056; Supplementary Fig. 2D). These results suggest that circulating EPCs and Tang cells are not significantly associated with ventricular SDF-1α or VEGF-α gene expression.

## Discussion

This is the first study to characterize circulating EPC and Tang cell counts alongside myocardial angiogenic factor in a clinically relevant HFpEF model. Thus, we provide a detailed phenotypic and angiogenic characterization of 38-week-old ZSF-1 obese rat as a model of severe HFpEF. Consistent with the clinical syndrome, these rats exhibit obesity, hypertension, diastolic dysfunction, and severe exercise intolerance, while maintaining preserved systolic function. Our findings strengthen the validity of this model for mechanistic studies of HFpEF and, more importantly, reveal a surprising dissociation between circulating and myocardial angiogenic profiles in severe HFpEF.

The hallmark of HFpEF pathophysiology is the combination of left ventricular stiffness, systemic inflammation, and microvascular dysfunction, rather than overt systolic failure. The observed increases in E/e′, E/A ratio, LVEDP and Tau confirm impaired ventricular compliance and relaxation in ZSF-1 obese rats. The preserved LV ejection fraction and fractional shortening further support a phenotype that mirrors clinical HFpEF, where structural and metabolic stress precede overt contractile failure. These findings are in tandem with previous reports of severe HFpEF in this model from 20 weeks of age^33,34^. The pronounced exercise intolerance provides functional validation of advanced disease severity at 38 weeks of age. Albeit we acknowledge that differences in body weight between ZSF-1 Obese and WKY rats may have influenced treadmill performance independently of cardiac dysfunction, particularly with reference to mechanical and/or orthopedic limitations occasioned by obesity as reviewed elsewhere^33,34^. In male and female ZSF-1 obese rats alike, HFpEF phenotype begins at 10 weeks of age and progressively worsen in disease severity as measured with echocardiography and exercise capacity test^21,22,26^. ZSF-1 obese rats have been reported to survive up to 42 weeks of age without mortality concerns^35,36^. Hence 38 weeks of age is ideal for measuring features of severe HFpEF in this model with hundred percent survival rate in our hands.

A striking observation in this study is the absence of significant differences in circulating EPCs and Tang cells between ZSF-1 obese and WKY rats. EPC and Tang cells contribute to vascular repair and neovascularization through paracrine and homing mechanisms^37,38^. Their comparable levels in both groups suggest that the circulatory capacity for endothelial regeneration remains intact, at least in the chronic phase of HFpEF in this model. However, the lack of compensatory increase in these pro-angiogenic populations despite evidence of myocardial inflammation implies a dysfunctional signaling between the heart and bone marrow. This angiogenic dissociation phenomenon may reflect impaired mobilization or defective recruitment of progenitor cells to the myocardium. Human HFpEF studies demonstrate that circulating EPC and Tang cell counts are significantly low when compared to healthy control^8,16,17^. Also, exercise regimen is reported to improve circulating EPC and Tang cell counts in HFpEF patients^8,39^ and this has been associated with improved nitric oxide signaling^39^. The discrepancy between our preclinical data and clinical reports which found significant decreases in EPC and Tang cell counts^8,16–18^ may not merely be species dependent. A different clinical study did not find significant differences in EPC counts between HFpEF patients and healthy control^19^. The inconsistency in reported EPC counts among HFpEF studies likely reflects population heterogeneity. Differences in EPC definition (e.g., CD34⁺/CD133⁺/VEGFR-2⁺ markers), and pre-analytical handling can yield divergent results. In addition, cohort composition, comorbidity burden, medication use, and variable disease stage and severity may influence EPC mobilization. Given the genetic and strain differences between ZSF-1 obese and WKY rats, the result of the present study is open to two interpretations. It is likely that severe HFpEF rats retain the normal capacity to produce EPCs and Tang cells from bone marrow or that the WKY control rats have inherently low baseline levels of these circulating angiogenic cells. One avenue to clarify these possibilities would be to conduct a comparative analysis of circulating angiogenic cell counts in WKY, ZSF-1 lean and ZSF-1 obese rats at 8, 18 and 38 weeks.

While it is plausible that regenerative capacity of circulating angiogenic cells may wane with age and disease progression, it is unknown if HFpEF severity/stage impacts circulating counts of EPC and Tang cells. The logical next step is to characterize the evolution of circulating EPC and Tang cells counts as well as myocardial angiogenic signaling in tandem with preclinical HFpEF progression and severity. To date, there exists no report comparing EPC and Tang cell counts by HFpEF disease severity in experimental models or human patients. Such reports will be crucial to confirm whether EPC and Tang cell population evolve with HFpEF disease progression and may potentially reveal a window of therapeutic target.

In contrast to the preserved circulating angiogenic markers, we observed a significant downregulation of myocardial SDF-1α and VEGF-α gene expression in ZSF-1 obese rats. Both factors are central to the maintenance of endothelial integrity and neovascularization. SDF-1α functions as a chemoattractant for progenitor cells and is a key regulator of stem cell homing via the CXCR4 pathway^40–45^, while VEGF-α drives endothelial cell proliferation and survival^46^. Their simultaneous reduction suggests a suppression of local angiogenic signaling within the hypertensive, metabolically stressed myocardium. This is consistent with prior reports of microvascular rarefaction and endothelial inflammation in HFpEF hearts^12,47^, which may be further exacerbated by reduced angiogenic transcriptional activity. We concede that significant reduction in the expression of genes does not directly imply reduction in their respective protein levels. Future endeavors should confirm the protein expressions and cellular sources of ventricular SDF-1α and VEGF-α. However, the lack of correlation between ventricular SDF-1α and VEGF-α genes and circulating angiogenic T cells suggests that peripheral mobilization of these cells may be regulated independently of local myocardial pro-angiogenic signaling. This finding implies that circulating cell counts may not serve as a reliable surrogate for myocardial angiogenic activity, highlighting the need to consider additional systemic or paracrine factors that govern angiogenic cell trafficking and function. Furthermore, the weak positive trend observed with VEGF-α, although not statistically significant, raises the possibility of subtle interactions that may emerge with increased sample size or under pathophysiological stress, warranting further investigation into the complex crosstalk between the myocardium and circulating progenitor cells.

Conversely, the significant upregulation of IL-1β in the left ventricle of ZSF-1 obese rats indicates activation of a pro-inflammatory milieu that likely antagonizes angiogenic signaling. IL-1β is a potent driver of endothelial dysfunction^48^, fibroblast activation^49^, and extracellular matrix deposition^50^, all of which contribute to ventricular stiffness. The concurrent downregulation of SDF-1α and VEGF-α in the presence of elevated IL-1β suggests that chronic inflammation may suppress reparative angiogenic pathways. Such interplay aligns with the emerging paradigm that HFpEF represents an inflammation-driven, endothelial-fibrotic continuum rather than a purely hemodynamic disorder.

Taken together, our findings support a model of severe HFpEF in which circulating angiogenic cell populations are quantitatively preserved but may be functionally ineffective in the HFpEF heart due to a hostile myocardial microenvironment characterized by inflammatory suppression of angiogenic signaling. This distinction between circulating and tissue-level angiogenic capacity may explain the limited regenerative responses observed in HFpEF and highlights the need for therapeutic strategies which may restore endothelial function and local angiogenic signaling, rather than merely augmenting circulating progenitor cells.

Our findings have important translational implications. Firstly, in experimental models of severe HFpEF, they suggest that circulating EPC or Tang cell counts alone may not reliably reflect myocardial angiogenic status, highlighting the need for tissue-level biomarkers in HFpEF. Secondly, therapeutic strategies aimed at restoring myocardial angiogenic signaling during pre-HFpEF or early diseases stage, rather than simply mobilizing circulating progenitor cells, may hold greater promise for mitigating HFpEF progression.

### Study limitations

Several limitations are herein acknowledged. ZSF-1 obese rats are genetically different from WKY rats, even though they share a common but distant lineage from the Wistar rats. The more appropriate genetically similar but hypertensive and weight control ZSF-1 lean rats would have been preferred to WKY. This study was conducted exclusively in male rats, and sex differences were not explored. Although HFpEF progression and severity are comparable between male and female ZSF-1 obese rats, angiogenic signaling may differ between sexes as ZSF-1 obese females are reported to develop type 2 diabetes later than males which exhibit significant hyperglycemia at 19 weeks^51^. Hence, our ongoing endeavor on ZSF-1 obese and lean rats of both sexes is set to confirm any sexual dimorphism in circulating and myocardial angiogenic markers. Our study captures a single timepoint of severe HFpEF. The present study reports a snapshot counts of EPCs and Tang cells in late-stage HFpEF model which limits insights into progenitor populations and potential early compensatory responses. Future studies should determine the longitudinal characterization and evolution of EPC and Tang cell count in relation to myocardial angiogenic signaling dynamics to provide temporal interpretation and insights for timely restorative therapeutic strategies. Whilst our sample size for both groups reached statistical significance for physiological differences, it is plausible than n = 6 WKY and n = 8 ZSF-1 obese male rats may be too small to detect true differences in circulating counts for EPC and Tang cells. Given that gene expressions do not necessarily reveal protein expressions, future reports on protein expressions of SDF-1α and VEGF-α along with colocalization histology would be informative in identifying cell types with these reduced expressions in addition to their respective gene levels. Myocardial capillary density and functional assays of circulating EPC or Tang cell angiogenic capacity were not performed; future studies are needed to directly link circulating cellular function with myocardial vascular remodeling.

## Conclusion

In summary, 38-week-old ZSF-1 obese rat recapitulates the hemodynamic, functional, and inflammatory hallmarks of severe HFpEF. Despite preserved systemic angiogenic cell counts, local cardiac angiogenic gene expression is markedly suppressed, coincident with increased inflammatory signaling. These findings suggest a pivotal shift from systemic angiogenic potential to myocardial angiogenic failure as a defining feature of advanced HFpEF in this experimental model. Protein expressions of the cardiac SDF-1α/VEGF-α axis and appropriate mechanistic studies may reveal a promising approach to restore microvascular integrity and improve diastolic function in HFpEF.

## Supplementary Information

Supplementary Information.

## Data Availability

Results of this study are available upon request.
