# Supplementary material for: Circulating pro-angiogenic cells are preserved despite myocardial angiogenic signal deficits in HFpEF model
Source: Sci Rep. 2026 Apr 14;16:17433. doi: 10.1038/s41598-026-47557-9 (PMC13237027; doi:10.1038/s41598-026-47557-9)
Supplement: Supplementary file 1 — Supplementary Information. [file 41598_2026_47557_MOESM1_ESM.pdf]

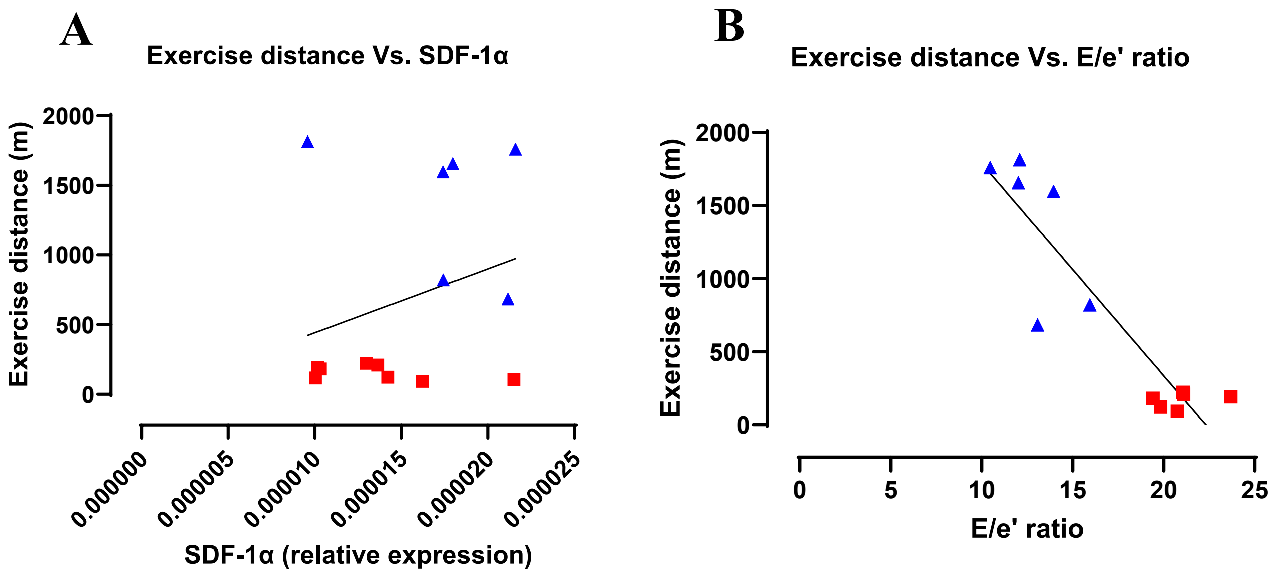

Supplementary Figure 1. Exercise is associated with SDF-1α but not ventricular filling pressures. (A) Correlation analyses plot between exercise distance and SDF-1α, (B) Correlation analyses plot between exercise distance and E/e’ ratio. * P<0.05. Blue triangles: Wistar Kyoto rats; Red squares: ZSF-1 Obese rats.

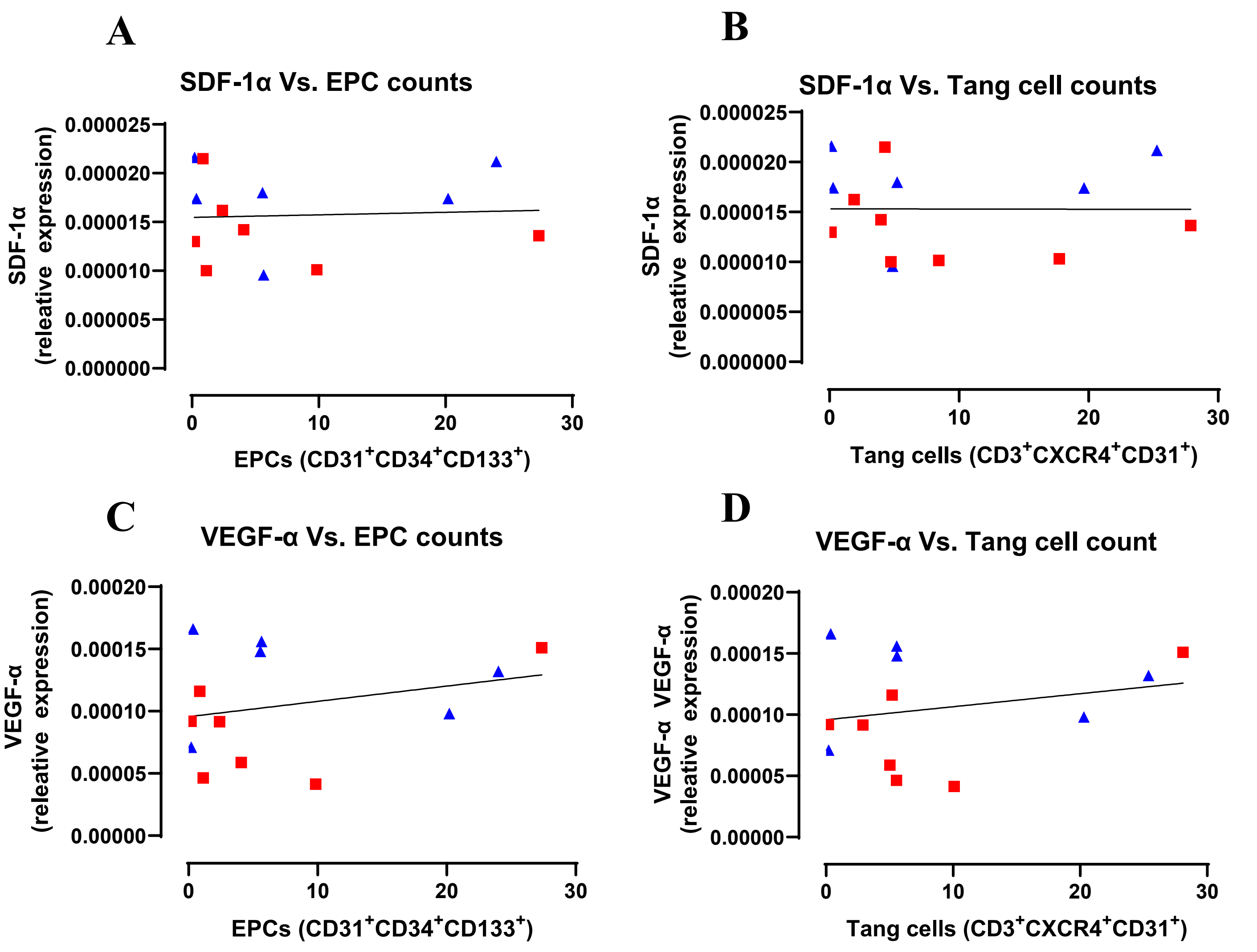

Supplementary Figure 2. Circulating angiogenic cell counts are associated with VEGF-α but not SDF-1α. (A) Correlation analysis plot between SDF-1α and Endothelial progenitor cells (EPCs), (B) Correlation analysis plot between SDF-1α and Tang cells, (C) Correlation analysis plot between VEGF-α and EPCs, (D) Correlation analysis plot between VEGF-α and Tang cells. Blue triangles: Wistar Kyoto rats; Red squares: ZSF-1 Obese rats.
